# Educational neighbourhoods in Vienna: A promising model to enhance educational opportunities and learning spaces through collaboration?

**DOI:** 10.1177/14782103251412478

**Published:** 2025-12-24

**Authors:** Nazime Öztürk

**Affiliations:** 1 Centre for Teacher Education, 27258 University of Vienna, Vienna, Austria

**Keywords:** inclusive education, educational neighbourhoods, collective responsibility, policy translation, stakeholder perspectives

## Abstract

Collaboration structures across relevant partners in education are associated with the potential to address contextual barriers to all learners’ presence, participation, and achievement. Following these ideas, a newly introduced educational policy in Vienna, the so-called *Bildungsgrätzl* (educational neighbourhoods), aims to foster long-term cooperative structures between schools and neighbourhood institutions (e.g. leisure facilities and communities). This study explored the perspectives of 14 local stakeholders, school leaders, and informal leaders on policy translation, following the criteria of classical grounded theory. By analysing the problem and solution representations provided by the participants, the study revealed several critical challenges within the educational system. These included structural issues, such as the operation of schools as isolated, closed systems; organizational challenges, such as the lack of effective collaboration among key educational stakeholders; and social factors, such as the underutilization of available opportunities. However, the findings indicate that collaboration is widely perceived as a promising response to the social inequities embedded in these settings, consistent with the initiative’s foundational goals. Furthermore, the results underscore relatively high expectations and assumptions about the role of *Bildungsgrätzl* in promoting inclusive education, despite the paradox of a highly segregated educational system in Austria that does not fully support these efforts.

## Introduction

In societies marked by migration and socioeconomic disparities, educational institutions are challenged to design equitable and sustainable systems for all learners (UNESCO, 2017). Inclusive education (IE) is increasingly recognized as a whole system approach in this endeavour, aimed at levelling the playing field and breaking down barriers in the presence, participation, and achievement of all learners (Ainscow, 2020). Accordingly, collective efforts of stakeholders and relevant actors within an education system are pivotal for creating an inclusive environment to enhance access to quality education and opportunities (Ainscow and Viola, 2023). To provide inclusive and equitable educational opportunities, supportive learning conditions that recognize academic and non-academic outcomes for all learners matter (Ainscow and Messiou, 2018; Felder, 2021). Studies have highlighted how collaboration between schools, local neighbourhoods (e.g. informal community organizations), and stakeholders can serve as a critical strategy for institutional improvement and foster students’ social and academic participation (Baldridge, 2023; Mowat, 2023). It is posited that collaboration among leaders at all levels committed to IE has the potential to increase local knowledge regarding contextual barriers to accessing educational opportunities and, thereby, contribute to their reduction (Ainscow, 2020).

Studies confirm that persistent inequities between learners regarding language, status, gender, and so on (OECD, 2017) and their intersections (Bešić, 2020) vary across countries, highlighting the importance of national contexts (Hardy and Woodcock, 2015). In Austria, social disparities are most pronounced at key educational transitions, where parental choice and school competition (Carroll and Walford, 1997) procedures often reinforce existing hierarchies (Öztürk, 2022). The Austrian education system is highly segregated, with over one-third of students diagnosed with special educational needs (SEN) placed in separate schooling (Buchner and Proyer, 2020). Moreover, the system is characterized by frequent transitions and entrenched hierarchies that shape students’ educational trajectories (Gitschthaler et al., 2021). Transition inequities to primary education in Austria occur in the interplay of social position (socioeconomic status of families) and local opportunities (resources at schools) on children’s educational pathways (Altrichter et al., 2014). The most significant separation between students arises after primary school, during the transition to either middle school (*Mittelschule*) or grammar school (*Gymnasium*), admission to which is contingent upon achieving a strong average grade. This early division reflects and perpetuates social inequities, as access to these educational pathways is heavily influenced by factors such as socioeconomic background and parental education (Avvisati et al., 2019). Further disadvantages have been proven for multilingual students, who fail to meet the expected language of instruction (German) level according to the School Organization Act (Neubacher and Wimmer, 2021). Additionally, students with a heritage language other than German are distributed differently in grammar schools (20.8%) and middle schools (34.8%), with trendsetting effects on their further academic careers (Statistik Austria, 2022). Furthermore, national policy programmes have a segregating impact on multilingual students who do not pass German language assessments during primary or secondary schooling (Öztürk et al., 2025). Nonetheless, the School Organization Act (§ 4) reflects a general commitment to inclusive values and policy promises in contradictory tensions to their implementation. In this sense, translation processes of political priorities regarding inclusive aims are much more complex in context, pervaded by different, sometimes competing, policy orientations (Paulsrud, 2022). Considering the possible gap between policy as text and delivering policy on the ground (Schulte, 2018), this study examines a newly introduced policy initiative claiming a significant shift towards IE by providing collaboration at the local level in Vienna.

### Vienna’s policy framework for collective responsibility towards inclusive education

In Vienna, educational neighbourhoods, the so-called *Bildungsgrätzl* (BG), were introduced in 2017 to foster long-term cooperative structures between schools, kindergartens, and other educational and leisure facilities within a district, such as youth work, libraries, and adult education centres (City of Vienna, 2024a). In this way, the idea of educational clusters (Brauckmann et al., 2018) or the Viennese approach of educational campuses (e.g. professional network systems between educational institutions) is translated to urban districts, and further non-formal institutions and communities are involved (Müller, 2020). The strategy’s implementation is backed by financial incentives from the City of Vienna. Currently, there are 34 accredited BG, receiving up to 5,000 euros annually, with the possibility of additional funding of up to 3,000 euros for specific projects (City of Vienna, 2024a). Since the implementation of BG is not mandatory and follows a top-down informed bottom-up logic, it is heavily influenced by the engagement and negotiations of practitioners (Ball, 2015). Additionally, this strategy is linked to the Sustainable Development Goals (SDGs) (United Nations, 2015) and is expected to promote equity, inclusion, and community well-being through collaborative educational efforts (Francesconi et al., 2024). The guiding principle, ‘It takes a Grätzl to raise a child’ (City of Vienna, 2024a), emphasizes the importance of community in taking shared responsibility for educational pathways. Recognizing that learning also occurs beyond traditional school settings, this collaboration seeks to foster open learning spaces that support children and young people throughout various educational stages – from early childhood education and schooling to the transition into the workforce (City of Vienna, 2024a). Therefore, efforts to increase the visibility of education and the impact of educational work through joint projects or activities (e.g. training for teachers, parent workshops, and professional learning support) are claimed to be relevant (Müller, 2020). The practical implementation of BG requires an exchange of information about joint content and pedagogical profiles (e.g. educational transitions, children’s rights, and multilingualism), where partners meet at regular intervals and share resources and competencies to find local responses (local agency) to perceived challenges (e.g. inequities) (Müller, 2020). However, key actors present ambivalent views by acknowledging the inclusive potential of BG on the one hand and stable structures that disproportionately benefit children from academically privileged families on the other (Schrott and Lener, 2020). At this point, there is no further research on how the policy of BG is translated and negotiated on the ground – the central question of this study.

### Collaboration as a pathway to inclusive education?

Theoretically, collaboration among schools, neighbourhood institutions, and stakeholders has been discussed over a long period as an effective strategy for broadening the capacity to respond to local diversity and needs (Ainscow and Viola, 2023; Chapman and Muijs, 2014; Hargreaves and Shirley, 2020; Ydesen and Daniels, 2024), especially in the US context (Green, 2015). Thus, building collaboration infrastructures across school neighbourhoods is linked to the potential to address contextual barriers to the presence, participation, and achievement of all learners as a shared responsibility (Ainscow, 2020). Collaborative networking among the wider community and local partners (caregivers, teachers, school administrators, etc.) is expected to provide insights into the unique challenges students face in accessing quality education and, thereby, increase possibilities for improvement at the institutional level (Hargreaves and Ainscow, 2015). According to Hargreaves and Shirley (2020), this approach can be defined as ‘leading from the middle’, as it promotes collaboration, initiative, and responsiveness to leverage the strengths and resources of various stakeholders for all students’ participation. However, studies have shown that the success of collaborative networking endeavours cannot be derived from their mere existence but depends heavily on their functionality (Chapman and Muijs, 2014) and supporting structures (e.g. sociopolitical landscape) within the wider educational system (Armstrong and Ainscow, 2018). Pooling resources, knowledge transfer, joint activities, and an orientation towards common goals with a focus on all students’ participation in the region have been described as pivotal aspects of functioning collaborations (Brauckmann et al., 2018). Additionally, financial support is crucial in fostering collective responsibility. Hargreaves and Braun (2011) found that extra financial resources enabled districts in Ontario to build a critical mass of support for inclusive reforms, while studies in England underscored how collaborative partnerships between high-performing and lower-performing schools resulted in students’ higher academic participation (Chapman and Muijs, 2014) and benefited particularly disadvantaged schools (Hargreaves and Ainscow, 2015). Besides these examples of school-to-school collaboration, the involvement of wider key actors and communities in the educational neighbourhood is expected to transform out-of-school challenges into opportunities (Green and Gooden, 2014). Additionally, findings underscore that community-based educational spaces (e.g. after-school programmes) can serve as third spaces and decentre schools as the main intelligible learning spaces (Baldridge, 2023). In addition to fostering intergenerational relationships and participation structures through shared responsibilities, studies have also outlined the role of cooperation in terms of its capacity to increase the visibility and value of formal education in the neighbourhood, contributing to its accessibility (Locatelli, 2024; Riley, 2013). Furthermore, the connection between the local context of learning and living has been addressed as promoting a sense of place and belonging (Yemini et al., 2023). As previously indicated, these positive expectations of scattered collaborative efforts for IE reach their limits if inclusive values are not shared widely in the education system (Ainscow, 2020). Moreover, studies have problematized how community-based approaches perpetuate deficit perspectives about some students and families, viewing them primarily as receivers rather than agents (Baldridge, 2023; Green and Gooden, 2014). However, such procedures hardly challenge power relations but perpetuate the status quo (Baldridge, 2014; Hardy and Woodcock, 2015). In light of the these findings, cooperative approaches in the education system are gaining traction (Armstrong and Ainscow, 2018) and can be evaluated as beneficial for promoting educational opportunities if supported structurally (Chapman and Muijs, 2014). At the same time, the prevalence of school-led approaches and enduring power imbalances within distributed leadership frameworks underscores the necessity for alternative governance structures. Without addressing these asymmetries, distributed leadership’s potential to foster collaboration, accountability, and systemic improvement remains limited (Papastephanou et al., 2020). To sum up, the possibilities and limits of collaborative efforts promising inclusion are linked to contextual dynamic and material practices. Further, stakeholders and key actors play a crucial role in enabling school–community relations and translating policy recommendations into practice (Green, 2017). So far, no studies exist regarding the unique case of BG implementation from the perspectives of key actors, and, in this respect, this study aims to fill this research gap.

## The present study

Considering the discrepancy between policy as text and its contextual enactment, which has been described as the ‘politics of use’ (Schulte, 2018), the current study examines the perspectives of stakeholders, school leaders, and informal leaders who shape and negotiate policy on the ground (Conn and Davis, 2024; Schulte, 2018). In doing so, the aim is to analyse educational problems and solutions represented by those responsible for and active in BG (Bacchi, 2017; Ball, 2015). The focus, therefore, is not on the given problems and proposals themselves but on how these issues are conceptualized, particularly concerning policy subjects (e.g. children and young people) in the view of the interviewees. However, portraying solutions to specific problems often reflects idealized visions – what Biesta (2023) refers to as ‘wishful thinking’ – about what is practically achievable. Taking these concepts into account, the following research questions guide the present study.

(1) What educational problems and solutions are represented by the stakeholders, school leaders, and informal leaders active in BG?
(2) What expectations and assumptions are ascribed to BG regarding the educational opportunities of children and young people?

## Materials and methods

### Participants and procedure

Access to the participants was secured through an ongoing project called ‘Teaching the Good Life. Theory, Policy and Practice in Education to Promote Quality of Life in the 21st Century’. A total of 14 individuals from seven different BGs participated in the study. The sample comprised six stakeholders, four school leaders from primary and secondary education, and four leaders from informal institutions, including those in adult education, social work, and environmental organizations (Table 1). Access was facilitated by the City of Vienna, which announced the project via email through BG networks. During the selection phase, some key actors were identified and named as potential participants. The project team reached out to these key informants and asked them to recommend additional participants with significant roles in planning, managing, and implementing BG. Additionally, institutional leaders within selected BGs were approached, the study was explained to them, and they were invited to participate. This approach ensured a diverse and comprehensive sample (Noy, 2008), following the principle of maximum variation (Corbin and Strauss, 2015). The interview guide included questions about the idea, implementation, organization, the role of education, and quality of life regarding BG. Semi-structured interviews according to Bogner et al. (2014) were conducted via Zoom (*n* = 9) or on site (*n* = 5). The interviews lasted 50.2 minutes on average (min: 39; max: 83). For this study, the quotes used from the interviews were translated from German into English and smoothed for readability (e.g. when dialects were present).

**Table 1.** Information on the participants.

Participant	Role in BG	Responsibility
S1	Stakeholder	School quality management
S2	Stakeholder	Coordination, founding, quality assurance
S3	Stakeholder	Coordination, founding, quality assurance
S4	Stakeholder	Training offerings for educational partners
S5	Stakeholder	Training offerings for educational partners
S6	Stakeholder	School quality management
SL1	Secondary school leader	Coordination, enactment
SL2	Primary school leader	Coordination, enactment
SL3	Primary school leader	Coordination, enactment
SL4	Primary school leader	Coordination, enactment
IP1	Informal partner	Coordination, funding application
IP2	Informal partner	Coordination, funding application
IP3	Informal partner	Coordination, workshop offerings for legal guardians
IP4	Informal partner	Coordination, funding application

### Ethical considerations

Prior to this study, ethical approval was obtained from the Ethics Committee of the University of Vienna (approval number: 01124). Interviewees gave their written consent to participate in the study, and the researchers assured all participants that sociodemographic information would be pseudonymized and data will be used solely for research purposes.

### Data analysis

The transcripts were analysed using MAQDA 2024 (VERBI, 2024) according to the criteria of classical grounded theory (Strauss and Corbin, 1994). In line with the methodological framework, the transcripts were openly coded to allow the emergence of patterns directly from data. Data were initially, axially, and selectively coded and analysed by constant comparison between codes, categories, and subcategories (Qureshi and Ünlü, 2020). Through the process, core categories emerged and formed the foundation for refining and saturating the concepts (Babchuk and Boswell, 2023). The analytical steps and findings of the author were discussed and double-checked by the project team to avoid biases as far as possible. The following interrelated categories were identified as assumed problem solutions to existing structures within the education system: opening educational institutions and opportunities, support for educational transitions, visibility of educational offers and spaces, and participation in the social space.

## Findings

### Opening educational institutions and opportunities

The BG initiative is conceptualized as a response to the structural problem of schools operating as isolated, closed systems with limited connections to other local institutions. By fostering collaboration among various institutions, BG aims to create a network that supports children, adolescents, and young adults throughout their educational trajectories. According to the participants, this approach is intended not only to enhance the educational success of students but also to optimize the functioning of participating institutions through mutual support and resource sharing. As one participant noted, ‘organizations can work optimally if they support each other’ (SL3). The potential of BG was seen in its capacity to reframe education as a collective responsibility, challenging the traditional siloed nature of schools. Interviewees emphasized that strategic cooperation among educational partners is essential for addressing systemic inequities and improving educational pathways. For instance, one participant highlighted the importance of joint planning, stating ‘if you approach education strategically, you also have to think about it together’ (S2). This collective approach was seen as a way to enhance institutional agency, enabling individual institutions to be more effective when operating within a collaborative framework (IP1, S2, S5, S6, SL2, SL3).

A key aspect of BG’s strategy is its focus on tailoring educational offerings to local needs. Examples include workshops for parents on children’s media use, which reflect an effort to bridge the gap between schools and the broader community (S2, S3, S6, SL1, IP1). By connecting different learning spaces – such as schools, homes, and public areas – BG seeks to decentralize schools as the sole dominant sites of learning. This decentralization aligns with the concept of lifeworld learning, where education is integrated into the everyday experiences of children and young people. Collaborative planning among BG partners was thus seen as a mechanism to open schools from being ‘closed systems’ and connect them to the social and cultural context of their communities (IP1, S3, SL2, S6).

The initiative’s broader goal is to address systemic inequities by fostering collective responsibility among educational institutions and other local organizations, including those involved in leisure education (IP2). This approach is particularly significant for children and young people who lack intensive support from their family contexts (S2, S3, S4, S6, IP2). As one participant observed, ‘it’s definitely an advantage if the players around the child move closer together’ (S5). By breaking down barriers between institutions, BG aims to reduce social inequities and promote inclusive opportunities for all students (S1, S2, S3, S5, S6, IP2). However, the interviews also revealed significant challenges to achieving these goals. While BG aspires to break down segregation dynamics in Austria’s education system, participants identified both internal (e.g. time and personnel constraints) and cross-institutional limitations (e.g. segregated transition structures) that hinder its implementation (S2, S3). These structural barriers underscore the tension between the initiative’s inclusive aspirations and the entrenched inequities of the broader system. Despite these challenges, BG was described as a network where ‘a joint effort is made to improve the educational success of children and young people and to contribute to equal opportunities in the long term’ (S2).

### Support for educational transitions

The interviewees highlighted networking between schools and other institutions as a critical mechanism for improving educational transitions, emphasizing its potential to enhance accessibility and streamline panning and monitoring processes for children (IP2, IP4, S1, S2, S3, S6). This approach reflects a strategic effort to address systemic barriers, such as dropouts, by ensuring that children at least secure apprenticeships rather than becoming unskilled workers (S1). Collaboration was seen as a way to mitigate the challenges of unfamiliar requirements during transitions, thereby reducing the risk of exclusion (S3).

Knowledge transfer, facilitated through teacher observations and BG networking meetings, was identified as a practical tool beneficial for aligning institutional efforts and ensuring seamless transitions (IP2, IP3, S1, S3, SL1, SL3). This underscores the importance of differentiation in fostering inclusivity, particularly for children with special needs, who might otherwise face significant setbacks when transitioning to new institutions (SL3). The emphasis on removing barriers aligns with broader goals of equity, enabling underrepresented groups to access in grammar schools and universities. For instance, changing perceptions about educational possibilities, such as attending university, was seen as a transformative outcome of these efforts (S5). Raising awareness of local educational offerings through BG activities was framed as a means to build trust and reduce transition-related hurdles for both children and their families (IP2, IP4, S1, S2, S3, S6, SL3).

And if there is already contact with these institutions, then of course there is a better chance that a child will follow such an educational path if they have the opportunity. Yes, and perhaps we can also provide support (SL3).

However, the assumption that visibility alone ensures participation risks oversimplifying the socioeconomic and cultural complexities involved. While some participants were optimistic about BG’s inclusive potential, others noted contradictions, such as school profiling prioritizing institutional interests over inclusivity (S3). It was mentioned that some schools seek to put their own profits in the foreground by advertising for supposedly better-performing student groups.

### Visibility of educational offers and spaces

The interviews linked socioeconomic disparities in accessing educational opportunities to a lack of information, emphasizing the need to make offers and spaces visible to enhance participation and reduce inequities (IP2, IP4, S3, S5, S6, SL1). Visibility was seen as particularly beneficial for non-German-speaking families and those with limited economic resources, as accessible, BG-financed, and free services could ease participation (IP2, S5). According to the interviewees, this would enhance students’ participation in education and reduce social inequities:

However, the opportunities are often not known and there are not always parents who can take their children across Vienna to a different support offer every afternoon. But of course, this is a minority and, for the others, these offers need to be visible in the BG (S5).

Extracurricular offers, such as youth services, learning support and park activities were expected to reach families with restricted access to full-day childcare options,^1^ helping them recognize and develop their own potential (SL3). Participants also highlighted that raising awareness of local opportunities could anchor educational efforts more firmly in public consciousness, fostering broader recognition of education’s value (IP1, IP4, S2, S6, SL1, SL2). However, the effectiveness of visibility in addressing deeper structural barriers remains implicit.

### Participation in the social space

The interviews emphasized BG’s role in fostering familiarity with public spaces to promote inclusion and safety for children and young people (IP1, IP2, IP4, SL2, SL3, S3, S6). This was linked to broader societal benefits, such as enhancing belonging and security through environments where people recognize and connect with one another (IP4). ‘Cross-institutional and cross-age work on a common theme’ (IP1) in the public space was assumed as a way to enrich academic and social experiences beyond formal institutions, though underutilization of spaces like parks was criticized, with children often ‘unsupervised or supervised by digital media’ (SL3). Participants argued that leveraging third spaces in BG could expand access to educational and leisure opportunities, such as learning support or sport activities, improving daily life and fostering collective responsibility, particularly for vulnerable groups like refugees and traumatized children (IP1, IP2, IP3, IP4, S3, S5, S6, SL2). The interviewees mentioned how insights into the ‘respective lifeworlds of others’ (IP2) would promote intergenerational understanding, trust, and social cohesion in BG (e.g. older people’s tolerance of the behaviour of children and young people) (IP1, IP2, IP3, IP4, S1, S6, SL1).

Participants’ quotes illustrated that collaboration within BG fosters pedagogical awareness of various local problems and inequities (e.g. poverty, loneliness, unsafe areas, and lack of German language), enabling shared responsibility in addressing and reducing social exclusion (IP2, SL2, S1, S6). For instance, providing professional translators was highlighted as a way to relieve multilingual students of the burden to act as translators for their families (IP2). Accordingly, participants stressed the importance of making support structures, such as ‘contact people [street workers] or contact points [libraries] outside the school’, visible to young people (IP1, IP3, IP4, S6, SL3, SL4). In this sense, networking with informal partners in BG was valued in its capacity to reach the age- and interest-diverse target group of children and young people (IP1). However, concerns were raised about the limited participatory involvement of young people in planning procedures, which is often dominated by BG leaders (IP1, IP2, IP4, S2, S3, SL3). A long-term vision was proposed to engage the target group ‘in a co-designing, co-producing, co-thinking way’ of their social spaces (SL4). This reflects a tension between top-down planning and the need for inclusive, participatory approaches.

## Discussion

This study evaluates the perspectives of stakeholders, school leaders, and informal leaders shaping and negotiating *Bildungsgrätzl* (BG), at the local level in Vienna. As the number of foundations has steadily increased since the initiative’s introduction, this first qualitative study provides insights into the expectations and assumptions of those responsible for planning, managing, and implementing BG. The results indicate that BG efforts are widely perceived as a promising response to social inequities, aligning with goals like fostering inter-organizational cooperation, inclusion, and third learning spaces (City of Vienna, 2024a). Respondents connected these goals to the unique local needs, thus contextualizing the initiative’s objectives and representing perceived educational problems and solutions. However, the discussion reveals a notable imbalance: Solutions are emphasized more than the underlying problems, which are illustrated only through a few examples. This reflects ‘wishful thinking’ in policy discourse, where rhetorical commitment to solutions may mask a lack of comprehensive problem analysis (Biesta, 2023). Such an approach aligns with Bacchi’s (2017) concept of problem representation, where solutions often presuppose implicit problem definitions, underscoring a potential gap in thoroughly addressing structural challenges. For example, the assumption that information transfer and visibility of opportunities via BG would result in active participation reproduces a rational choice logic, according to which being informed successfully leads to social mobility (Carroll and Walford, 1997). Papastephanou et al. (2020) critique the reduction of justice to singular dimensions, emphasizing that distributed leadership often overlooks epistemic and restorative justice, both essential for addressing systemic inequities in education. Indeed, realizing the transformative potential of distributed leadership requires a multidimensional understanding of justice that considers diverse contexts and claims. The perspectives of BG key actors (Schrott and Lener, 2020), as well as hints in the data, point to a paradoxical direction: Although not intended by the policy (Hall et al., 2024), various mechanisms such as school profiling (Altrichter et al., 2014) and parental choice procedures further contribute to inequities during transitions (Öztürk, 2022), contradicting BG’s efforts to promote IE (Schrott and Lener, 2020). In this respect, the availability of educational opportunities does not guarantee access to quality education if contextual factors are not recognized in its interplay in policymaking and evaluation. This raises critical questions about how intensively BG activities are organized and whether they reach their addressees.

Regarding the perceived problems within the educational system, the results reveal several challenges that are believed to be addressed via BG: Structural issues like schools operating as isolated systems, organizational challenges, such as the lack of effective collaboration among stakeholders, and social factors like the underutilization of available opportunities. Although several different educational partners are addressed in the policy to mobilize resources, the examples given in the material point in a school-led direction (Armstrong and Ainscow, 2018). This raises questions about the extent to which distributed leadership, as a theoretical framework, is genuinely operationalized in BG. While distributed leadership is often associated with shared responsibility and collective agency, the findings suggest that cultural and institutional contexts in Austria may limit its application. This case thus challenges dominant understandings of distributed leadership by highlighting how policy and governance frameworks shape its enactment in distinctive ways.

Additionally, participants criticized inadequate collective responsibility for the educational pathways of students at risk of encountering barriers due to language, poverty, and SEN (Avvisati et al., 2019; Buchner and Proyer, 2020; Neubacher and Wimmer, 2021). Expected solutions were highlighted as directly linked to the capacity of BG. Given the complexities of educational practice (Biesta, 2023) and the resilience of education systems (Felder, 2021), the sphere of influence of BG seems to be limited, conflicting with the high expectations of the participants, for example, to challenge the segregation dynamics inherent in the education system of Austria (Gitschthaler et al., 2021). In particular, holistic claims such as fostering social cohesion and reducing social inequities can be related to an ‘educationalization of social problems’ concept (Tröhler, 2016), implying that societal problems are framed as solvable through appropriate educational strategies and policies. Beyond that, the scope for individual measures is interdependent on further different and coexisting political priorities in an education system (Magnússon et al., 2019; Paulsrud, 2022). To strengthen the practical implications of this study, institutions could adopt strategies such as fostering cross-institutional trust regular joint planning sessions, creating shared accountability frameworks, and providing targeted professional development for teachers and institutional leaders to enhance their capacity to implement inclusive practices.

In the Austrian context, concurring policy ideals coexist, and practitioners (school leaders, teachers) are required to apply segregating measures, such as language proficiency tests, which for some multilingual children mean special lessons separate from the mainstream class (Öztürk et al., 2025), while compensating for disadvantages that arise (e.g. social exclusion), especially in the case of BG advocating for social belonging (Yemini et al., 2023). Furthermore, the assumptions to support transitions in changing imaginations about possible futures (e.g. attending a grammar school or starting an apprenticeship) within families lacking socioeconomic privileges must be balanced with counteracting forces of segregated transitional structures of the Austrian education system. Under these conditions, BG functions as a counterstrategy, aiming to decouple academic potential from personal and social circumstances. However, to this end, a general commitment is needed in BG and at all levels of the education system (Ainscow, 2020). In this context, the interviewees sporadically reference cross-institutional restrictions that underline the awareness of BG’s limits.

Participants expect the cooperation between the institutions to have a multiplier effect, enhancing the capacity of the institution’s agency (via knowledge transfer, joint planning, and joint activities) to respond to the specific needs of children and young people more effectively (Hargreaves and Shirley, 2020). For example, in line with research findings (Chapman and Muijs, 2014; Ydesen and Daniels, 2024), informal partners’ insights into the diverse lifeworlds of children and young people are evaluated as crucial for recognizing and addressing social exclusion mechanisms (e.g. by providing translators). These findings suggest that distributed leadership, when supported by actionable strategies, could enhance the functionality of BG and its capacity to address systemic inequities.

Furthermore, the results affirm a relatively clear understanding of the students who may be at risk of being left behind, specifically those from socioeconomically disadvantaged backgrounds or refugee families, or those with special educational needs (SEN). This suggests that these groups encounter structural disadvantages in Austria, which are compounded by implicit low academic expectations and reinforce fixed notions of ability (Baldridge, 2023; Conn and Davis, 2024). In this sense, Baldridge (2023) draws attention to the situatedness of community-based educational work, bringing it into the same contextual structures as schooling and, thus, making a sustainable infrastructure for agency more important to be able to lower opportunity gaps. Described ways of opportunity provision, by showing structures (visible socializing and learning spaces) and providing out-of-school experiences with the help of financial support from the city of Vienna, may raise awareness about collective responsibility for students’ social and academic participation. Based on the findings, it can be inferred that a certain degree of functionality exists (Hargreaves and Braun, 2011). However, the extent to which resources are fully utilized remains unclear, as does the level of commitment exhibited by teachers – who play a crucial role in translating the BG – compared to the participants in this study.

Regarding the participation of children and young people, the initiative represented by the key actors builds heavily on social belonging and trust (feeling included and trusting the people and institutions) (Felder, 2021) as a precondition for levelling barriers to access to institutions and quality education. Giving consideration to the interrelated spaces of learning and living in educational efforts has been discussed as a promising way to promote IE (Ydesen and Daniels, 2024). According to the data, there were some hints that information transfer between informal partners, with their emphasis on community engagement, and school leaders, with their focus on resource allocation, might benefit the educational processes of children and young people. However, further questions regarding the material practices and dynamics to support experiences of inclusion or exclusion remain unanswered and need to be addressed in future research. By framing these insights as actionable strategies, such as fostering participatory planning processes and embedding inclusive leadership practices, the study could provide a stronger foundation for practical applications in policy and leadership contexts.

## Conclusion

As part of the broader policy framework (United Nations, 2015) to promote quality education, BG represents a (g)locally adaptable model where stakeholders actively shape policies at the neighbourhood level. Considering possible gaps between envisioned policies and their enactment (Schulte, 2018), this study explored the views of key actors who interpret and adapt policy within local conditions. The study provides insights into the contextual possibilities and limitations of policy implementation. Even if the policy of BG offers new and valuable impulses for collaborative education management to enhance educational opportunities, its implementation remains relational to structural conditions, the coexisting concurrent political demands of the education system, and the attribution of meaning by practitioners. Thus, Austria is a good case for examining the different aspirations of educational policies and translations in the respective district environment. Given that implementing BG relies heavily on key actors’ commitment rather than mandatory policies, these voices are valuable resources for policy evaluation and development that align with the principles of IE (Ainscow, 2020).

## Limitations

A substantial limitation of the study concerns the sample composition, which consisted of people entrusted with relevant tasks regarding BG’s implementation, monitoring, designing, and process support and, thus, showcased a very high level of commitment. Although these insights were essential for analysing BG’s administrative structures, other perspectives remain important to generate further insights. For example, by accessing the fieldwork, it has become apparent that some school leaders are gradually deciding against implementing BG due to a claimed lack of resources (e.g. time and personnel). Furthermore, it became apparent that teachers play important roles in shaping out-of-school activities. Their voices should be explored in future research to gain more insights into BG’s functioning.

## Note

1. Access to a free full-day place at a public elementary school is primarily intended for working legal guardians, even though there are also paid after-school care options at public elementary and secondary schools in Vienna. Further, access to these formats is heavily influenced by structural (available places) and familiar decisions (City of Vienna, 2024b).
